# Determination of Branched-Chain Keto Acids in Serum and Muscles Using High Performance Liquid Chromatography-Quadrupole Time-of-Flight Mass Spectrometry

**DOI:** 10.3390/molecules23010147

**Published:** 2018-01-11

**Authors:** You Zhang, Bingjie Yin, Runxian Li, Pingli He

**Affiliations:** State Key Laboratory of Animal Nutrition, College of Animal Science and Technology, China Agricultural University, Beijing 100193, China; you_93@cau.edu.cn (Y.Z.); youda_93@163.com (B.Y.); 18883870725@163.com (R.L.)

**Keywords:** branched-chain keto acids, serum, muscle, HPLC-Q-TOF/MS

## Abstract

Branched-chain keto acids (BCKAs) are derivatives from the first step in the metabolism of branched-chain amino acids (BCAAs) and can provide important information on animal health and disease. Here, a simple, reliable and effective method was developed for the determination of three BCKAs (α-ketoisocaproate, α-keto-β-methylvalerate and α-ketoisovalerate) in serum and muscle samples using high performance liquid chromatography-quadrupole time-of-flight mass spectrometry (HPLC-Q-TOF/MS). The samples were extracted using methanol and separated on a 1.8 μm Eclipse Plus C18 column within 10 min. The mobile phase was 10 mmol L^−1^ ammonium acetate aqueous solution and acetonitrile. The results showed that recoveries for the three BCKAs ranged from 78.4% to 114.3% with relative standard deviation (RSD) less than 9.7%. The limit of quantitation (LOQ) were 0.06~0.23 μmol L^−1^ and 0.09~0.27 nmol g^−1^ for serum and muscle samples, respectively. The proposed method can be applied to the determination of three BCKAs in animal serum and muscle samples.

## 1. Introduction

The branched-chain amino acids (BCAAs) leucine, isoleucine, and valine are three essential amino acids that cannot be synthesized by animals and must be obtained from foods [1,2,3]. As a result of the stimulatory effect of BCAAs on protein synthesis and direct effects on glucose transport and insulin secretion, investigations into the metabolism of BCAAs have become a popular research area in recent years, with particular emphasis on the branched-chain keto acids (BCKAs) [4,5,6,7,8,9,10]. The BCKAs are derived from the initial step in the catabolism of BCAAs via BCAA amino transferase, which transfers the amino group of BCAAs to α-ketoglutarate to form the BCKAs (including α-ketoisocaproate (KIC, ketoleucine), α-keto-β-methylvalerate (KMV, ketoisoleucine) and α-ketoisovalerate (KIV, ketovaline)) in the muscle [11,12,13]. After BCAAs are catalyzed by amino transferase, BCKAs can be irreversibly oxidized to isovaleryl-CoA, isobutyryl-CoA and 2-methylbutyryl-CoA via the enzyme branched-chainketo acid dehydrogenase (BCKDH) [14,15,16]. The effect of BCKAs on stimulating protein synthesis has been reported [17,18]. And when the BCKDH complex is mutated and defective, the BCKAs will accumulate in tissues resulting in development of the maple syrup urine disease (MSUD) in animals [19]. Since BCKAs have such an important effect on the metabolic and nutritional state of animals, accurate determination of BCKAs in biological samples is highly valuable.

Several analytical methods have been applied to measure BCKAs in biological fluids such as plasma and urine. These include gas-liquid chromatography (GC) [20], gas chromatography-mass spectrometry (GC-MS) [21] and high performance liquid chromatography (HPLC) with fluoresce detection [22,23,24]. However, these techniques are either time consuming or lack sensitivity such that certain BCKAs are not detectable in tissue. Ultra-fast liquid chromatography-mass spectrometry (UFLC-MS) methods are quite sensitive with shorter analysis times and have become popular for the detection of keto acids. Nevertheless, this method relies on the similar derivatization of keto acids with *o*-phenylenediamine (OPD), which results in a tedious preparation procedure and detrimental exposure to toxic reagents [25].

In this study, we developed a simple and accurate method to determine the three BCKAs in serum and muscle samples using high performance liquid chromatography-quadrupole time-of-flight mass spectrometry (HPLC-Q-TOF/MS). Samples are extracted by methanol which can remove protein efficiently and the extracted solution was concentrated to improve the detection sensitivity. The high resolution mass spectrometry (HRMS) with high selectivity and resolution assured the accuracy of the results. The wide range of linearity with reproducibility over a broad range of concentrations makes it a convenient way to determinate BCKAs in serum and muscle samples. Compared with previous research [25], it could been seen that when multiple samples need to be analyzed, the method we described can considerably simplify pretreatment procedures.

## 2. Results and Discussion

### 2.1. Optimization of HPLC Q-TOF/MS Conditions

**Figure 1:**
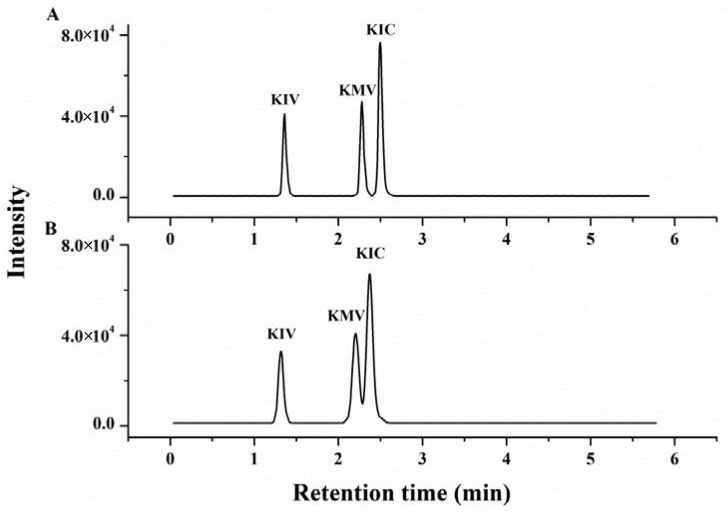
Influence of different columns on the HPLC performance of the branched-chain keto acids (KIV: α-ketoisovalerate; KMV: α-keto-β-methylvalerate; KIC: α-ketoisocaproate) mixed standard solution (2.5 μmol/L for each compound): (**A**) Agilent Eclipse Plus C_18_ column; (**B**) Waters BEH C_18_ column.

Chromatographic conditions such as analytical column, mobile phase composition and injection volume were optimized to obtain better performance on separating target compounds in standard solution and sample matrix. For example, the Waters BEH C_18_ (2.1 × 100 mm, 1.7 μm) column and the Agilent Eclipse Plus C_18_ (2.1 × 100 mm, 1.8 μm) column were compared to separate the three BCKAs. From Figure 1 we can see that, the Agilent Eclipse Plus (2.1 × 100 mm, 1.8 μm) C_18_ column was more suitable for separating the KMV and KIC in a 2.5 μmol/L standard solution for each compound, with better overall peak shapes and a better separation displayed by the resolution (>1.5 for KMV-KIC). Therefore, the Eclipse Plus C_18_ was chosen as the analytical column. In order to obtain an excellent separation and ionization effect, different concentrations (5, 10 and 20 mmol/L) of ammonium acetate were tested. A previous study had showed that the 10 mmol/L aqueous ammonium acetate improved the separation of the three BCKAs better than the lower concentration [26]. The 10 and 20 mmol/L ammonium acetate aqueous had similar performance in the separation and peak response of the three BCKAs. Considering the high concentration of ammonium acetate damaged the chromatographic column, the 10 mmol/L concentration of ammonium acetate was selected as the final aqueous phase. At the same time, in order to sufficiently elute the samples, we chose a long elution procedure with gentle change of gradient for this technique.

**Figure 2:**
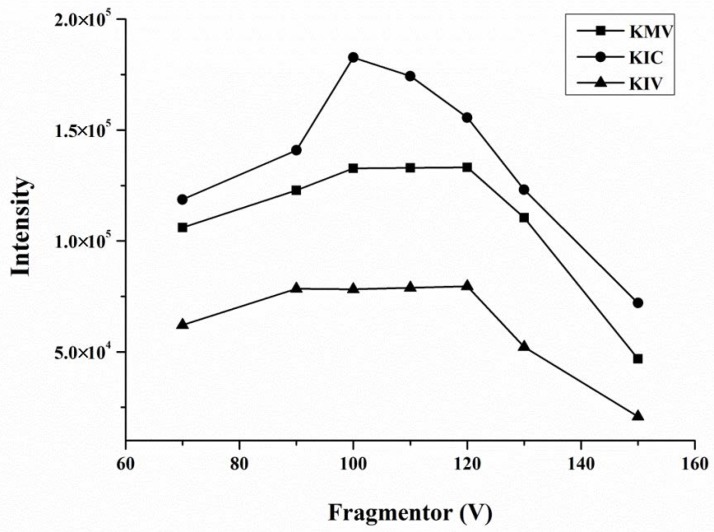
Intensity of 3 branched-chain keto acids (KIV: α-ketoisovalerate; KMV: α-keto-β-methylvalerate; KIC: α-ketoisocaproate) mixed standard solution (5 μmol/L for each compound) in different fragmentor (V).

The optimization of mass spectrometry parameters included capillary and fragmentor voltages, nebulizer pressure and collision energy. Among these, fragmentor voltages had a dominant influence on the sensitivity of the compounds detection. By comparing the intensity of three BCKAs mixed standard solutions in different fragmentor voltages from 70 to 150 V, we determined that the three BCKAs had the maximum peak response when the fragmentor voltages were set at 100 V (Figure 2).

**Figure 3:**
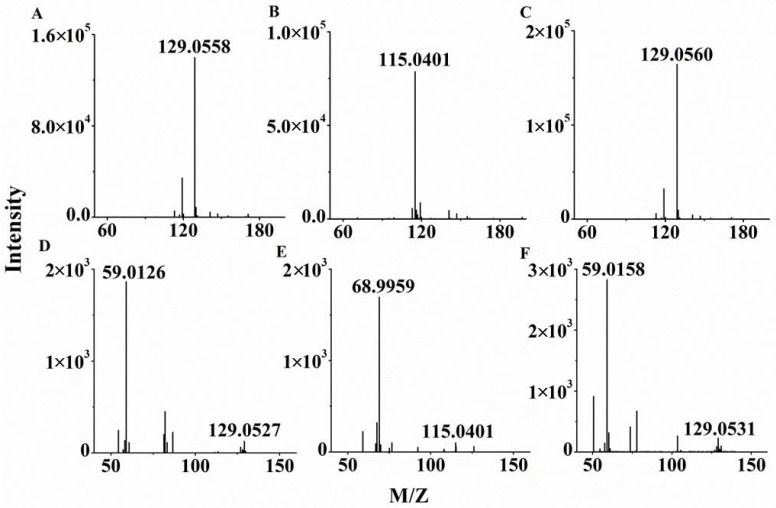
The mass spectra of the branched-chain keto acids standards using HPLC-Q-TOF/MS. (**A**) Full scan mass spectra of α-keto-β-methylvalerate (KMV); (**B**) Full scan mass spectrum of α-ketoisovalerate (KIV); (**C**) Full scan mass spectrum of α-ketoisovalerate (KIC); (**D**) MS/MS spectrum of KMV; (**E**) MS/MS spectrum of KIV; (**F**) MS/MS spectrum of KIC.

The collision energy was tested at 10, 15, 20, and 25 V, and it showed that 20 V was most appropriate for the three BCKAs, so the collision energy was set as 20 V. Under these conditions, the mass spectra of three BCKAs standards in HPLC-Q-TOF/MS were presented in Figure 3, including full scan mass spectra (Figure 3A–C) and MS/MS spectrums (Figure 3D–F). The retention time of three BCKAs and the fragment ions *m*/*z* were presented in Table 1, which were used for characteristic determination.

### 2.2. Optimization of Pretreatment Conditions

**Figure 4:**
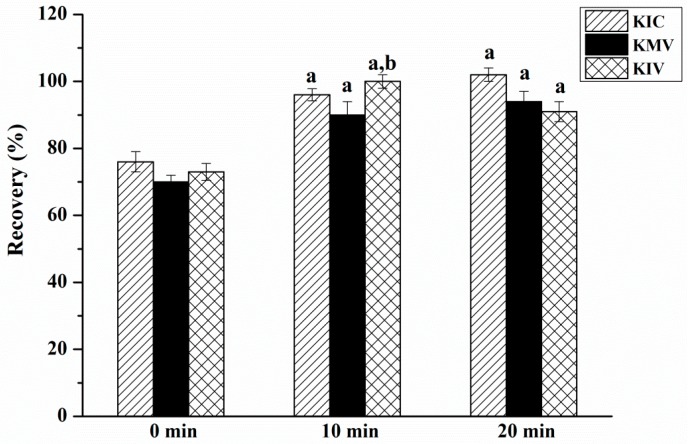
The influence of different ultrasonic time on the recoveries of three BCKAs (KIV: α-ketoisovalerate; KMV: α-keto-β-methylvalerate; KIC: α-ketoisocaproate). ^a^, ^b^ Means the same compounds different significantly (*p* < 0.05).

To accurately determine the three BCKAs in serum and muscle samples, an efficient extraction procedure is necessary. In this study, three extraction solvents including methanol, acetonitrile and methanol-acetonitrile (50:50, *v*/*v*) were compared. The results showed that most of the interfering proteins present in the two matrices were precipitated in the presence of methanol and can be efficiently removed by the subsequent refrigerated centrifugation procedure [26]. Thus, methanol was chosen as the extraction solvent. Figure 4 suggests that the recoveries were relatively lower when the samples were extracted without ultrasonic treatment. As a result, at least a 10-min ultrasonic treatment was required. In addition, drying of the extracted supernatant fluid and re-dissolution of the residues in a small amount of ultra-pure water removed organic solvent and concentrate samples, which improved the detected sensitivity to a large extent.

### 2.3. Method Validation

The sensitivity, accuracy and precision of an analysis method are usually validated using linearity, LOQ and recoveries of spiked samples. In this study, the HPLC-Q-TOF/MS-based method for the detection of three BCKAs showed satisfactory linearity within the concentration range of 0.1 to 100 μmol L^−1^ (R^2^ > 0.998), which covered the range commonly observed in serum and muscle samples. The LOQ is defined as the concentration at which the signal-to-noise ratio is more than ten. Because the BCKAs are endogenous compounds, it is difficult to obtain serum and muscle samples free of them. Hence, the blank samples were surrogated by other artificial matrices. Often, phosphate buffered saline (PBS) is used for serum analyses because of its pH (7.4) and ionic strength. Bovine serum albumin (BSA) is frequently added to PBS to take the protein content of biological matrix into account [27]. In this study, the serum and muscle samples were surrogated by 2% BSA in PBS and 1 g/L of BSA, respectively. Table 2 showed that the LOQ of the three BCKAs ranged from 0.06 to 0.23 μmol L^−1^ and 0.09 to 0.27 nmol g^−1^ for serum and muscle samples, respectively.

**Figure 5:**
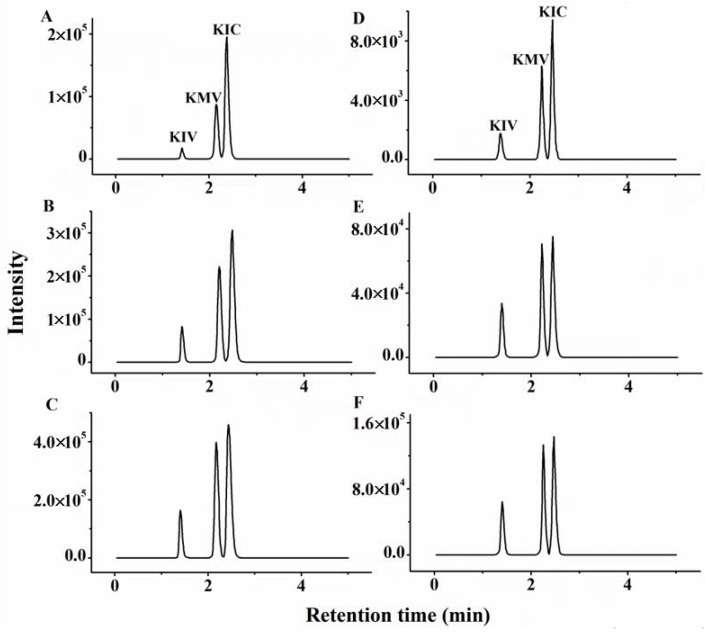
Representative chromatograms of BCKAs in different samples. (**A**) No-spike serum sample; (**B**) Low-spike serum sample; (**C**) High-spike serum sample; (**D**) No-spike muscle sample; (**E**) Low-spike muscle sample; (**F**) High-spike muscle sample.

Six replicates were tested for evaluation of spike recovery and relative standard deviation (RSD). The actual spiked concentration in the different matrices showed good consistency, and the calculated recoveries for three BCKAs when different concentrations of spiked solutions were used ranged from 78.4% to 114.3% with the RSD less than 9.7% (Table 2). The representative chromatograms of three BCKAs in no-spike, low-spike and high-spike serum and muscle samples are presented in Figure 5. The results showed that the BCKAs concentrations in the samples were all above the LOQ.

### 2.4. The Analysis of Authentic Sample

**Figure 6:**
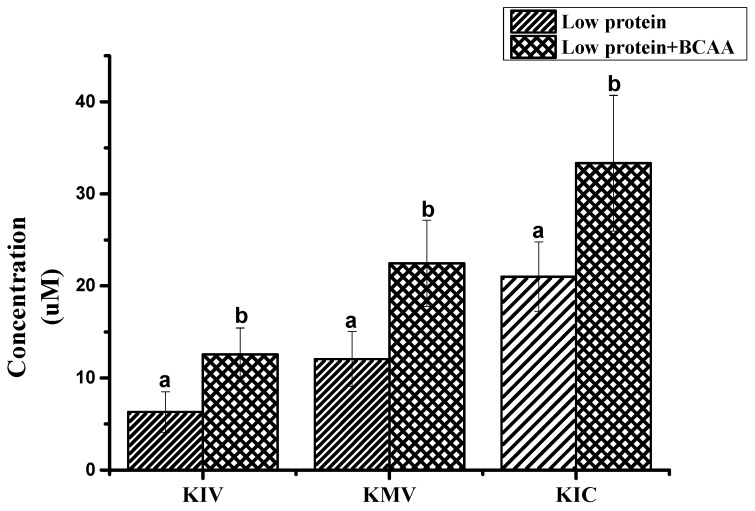
The influence of the addition of BCAA on the concentration of three BCKAs in serum of pigs fed different levels of dietary BCAA (KIV: α-ketoisovalerate; KMV: ketoisoleucine; KIC: α-ketoisocaproate). ^a^, ^b^ Means the same compounds different significantly (*p* < 0.05).

The proposed method was adopted for the determination of three BCKAs in authentic serum and muscle samples from pigs. Table 3 shows the concentrations ranges of three BCKAs in different samples, the reported variability is likely due to inherent variation among individual pigs and different levels of BCKAs in the diet. Nevertheless, the HPLC-Q-TOF/MS results were in good accordance with literature values, in which the concentration is about 20 nmol/L of KIV and KMV, 30 nmol/L of KIC [28,29,30]. Furthermore, we chose samples from pigs fed the 17% crude protein and 17% crude protein + BCAA diets which had significant differences in free AA concentrations of threonine, methionine, lysine and BCAA [9] to test the concentrations of three BCKAs in serum (*n* = 12). The results show that the addition of BCAA significantly increased the concentration of three BCKAs in precaval venous blood samples (Figure 6). The assay was able to detect differences in three BCKAs levels that reflected the differences in dietary BCAA supply, which demonstrates the availability and validity of the developed method.

## 3. Materials and Methods

### 3.1. Materials and Reagents

The HPLC grade three BCKAs and the corresponding internal standard [^13^C]-ketoisocaproate ([^13^C]-KIC) were purchased from Sigma (St Louis, MO, USA). Ultra-pure water purified using a Milli-Q system (Millipore, Bedford, MA, USA) was used to prepare all aqueous solutions. HPLC grade methanol and acetonitrile were obtained from Fisher Scientific International (Hampton, NH, USA). HPLC grade ammonium acetate was purchased from Dikma Technology (Richmond Hill, ON, Canada). Nylon membrane filters (0.1 μm) were obtained from Whatman (Maidstone, UK).

### 3.2. Apparatus and Procedures

The chromatographic separation was developed using an Agilent 1290 Infinity HPLC system coupled to a 6520 quadrupole-time of flight mass spectrometer (Q-TOF/MS) from Agilent Technologies (Santa Clara, CA, USA). An ultrasonic cleaner (Kunshan Ultrasonic Instrument Co., Kunshan, China) was used for promoting the sample dissolution and a vacuum concentrator (Eppendorf, Hamburg, Germany) was used to dry the supernatants.

### 3.3. Preparation of Standard Solutions

All standard stock solutions (10 mmol L^−1^) were prepared in ultra-pure water and stored at −20 °C until use. A mixture of three BCKAs standard stock solution was prepared by mixing and diluting the single stock solutions at 1 mmol L^−1^ and stored at −20 °C until use. Working standard solutions were freshly prepared daily by diluting the standard stock solution with ultra-pure water to five different concentrations (from 0.1 to 100 μmol L^−1^).

### 3.4. Sample Preparation and Extraction

Serum sample extraction was achieved by adding 800 μL methanol into 200 μL sample, then 4 μL of internal standard (0.5 mmol L^−1^ [^13^C]-KIC) was added. Samples were put under vortex movement for 1 min, deproteined for 2 h at −20 °C, and centrifuged at 13,000 rpm for 10 min at 4 °C. Collection of 500 μL supernatant fluid was evaporated to dryness in a vacuum concentrator. The residues were resuspended in 100 μL of ultra-pure water, vortexed, and centrifuged again at 13,000 rpm for 10 min at 4 °C. The supernatant passed through 0.1 μm syringe filter was transferred to a conical insert in the sampler vial and then used for HPLC Q-TOF/MS analysis.

Muscle samples were crushed in liquid nitrogen. Sample extraction was achieved by adding 900 μL extraction solution (methanol:water, 8:2, *v*:*v*) into 100 mg muscle samples and 4 μL of internal standard (0.1 mmol L^−1^[^13^C]-KIC) was also added. Samples were put under vortex movement for 1 min, then placed in an ultrasonic bath for 10 min to achieve full extraction. Then deproteination, supernatant purification and HPLC Q-TOF/MS analysis followed the same procedure as for the serum samples.

### 3.5. HPLC Q-TOF/MS Conditions

Chromatographic separation of three BCKAs was achieved on an Agilent Eclipse Plus C_18_ column (2.1 × 100 mm, 1.8 μm). The column temperature was set at 30 °C and the flow rate was 0.3 mL/min. The mobile phase was 10 mmol/L ammonium acetate aqueous (solvent A) and acetonitrile (solvent B). The compounds were eluted with a linear gradient, consisting of 5–30% B over 0–3 min, to 90% B at 3.5 min, the constant gradient at 90% B for 3 min, and 5% B at 7 min for 3 min (re-equilibration of the column) before the loading of the next sample, so the total run time was 10 min. The injection volume was 5 μL.

The separated components from HPLC were subsequently analyzed by Q-TOF/MS, which was in line with the HPLC and operated in an electrospray ionization negative mode. The settings were as follows: drying gas temperature at 350 °C, a desolvation flow rate of 12 L/min, and a nebulizer pressure of 60 psig. The capillary and fragmentor voltages were set at 3500 and 100 V, respectively. Data were acquired in a full scan mode (*m*/*z* 70–300) at the rate of 2 spectra/s. To ensure the mass accuracy of detected ions, reference molecules (*m*/*z* 121.050873 and 922.009798) were continuously introduced into the electrospray ionization source to perform internal calibration. The MS/MS spectra of the compounds were recorded at a rate of 800 ms/spectra with the collision energy in 20 V. The characteristic fragment ions were used for confirmation.

### 3.6. Validation Procedure

Method validation was performed with serum and muscle samples, including linearity, sensitivity, as well as recovery and accuracy of the three BCKAs in six replicates. The recoveries were determined by spiking samples with a mixture of BCKA standards at 10, 20, 50 μmol L^−1^ in serum or 1, 2, 5 nmol g^−1^ in muscles. The internal standard solution was added in both spiked samples and standard solution. The concentration of mixed standard solution was gradually diluted and analyzed in order to measure the limit of quantitation (LOQ, S/N = 10). The *m*/*z* of the 3 precursor ions were presented in Table 1 which were used for quantitative determination.

### 3.7. Analysis of Actual Animal Samples

Animals breeding experiments were carried out at Huazhong Agricultural University (Wuhan, China) as previously described [9]. All piglets used in this study were housed and handled according to the established guidelines of Huazhong Agricultural University. All procedures performed on the animals were approved by Huazhong Agricultural University Animal Care and Use Committee (approval permit number 30700571). After the experiments, blood samples from precaval veins were collected after fasting 12 h and centrifuged (3000× *g*) for 10 min. Then the supernatant was transferred into new tubes and stored at −20 °C. After blood was collected, pigs were slaughtered and longissimus dorsi was collected and stored at −20 °C until use.

### 3.8. Statistical Analysis

Data were analyzed using the analysis of variance (ANOVA) procedure of SAS system (version 9.2; SAS Institute Inc., Cary, NC, USA). A *p* value less than 0.05 was considered statistically significant.

## 4. Conclusions

The objective of this study was to develop a simple method to measure BCKAs in serum and muscle samples without derivatization. The HRMS offered excellent selectivity under complex matrices and ensured the collection of high quality data and the accuracy of quantitative data. The wide range of linearity of the assay exceeded the levels that were commonly observed in serum and muscle samples. Meanwhile, the LOQ of 0.06~0.23 μmol/L and 0.09~0.27 nmol/g for serum and muscle samples meets the generally desired quantifiable levels in physiological samples. The recoveries for the three BCKAs range from 78.4% to 114.3% with RSD less than 9.7%, which demonstrates the accuracy and precision of this procedure. Furthermore, the developed method has been validated using authentic serum and muscle samples. Thus the study provides a simple, reliable and effective method for the determination of three BCKAs in serum and muscle samples using HPLC Q-TOF/MS and the proposed method can be applied to the determination of three BCKAs in actual serum and muscle samples.

## Figures and Tables

**Table 1:** Chemical structure and tandem mass spectrometry parameters of the branched-chain keto acids.

Compounds	Molecular Formula	Structure	Retention Time (min)	Precursor Ion (*m*/*z*)	Fragment Ion (*m*/*z*)
α-ketoisocaproate	C_6_H_10_O_3_	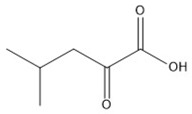	2.48	129.0542 (3.17) ^a^	59.0158
α-keto-β-methylvalerate	C_6_H_10_O_3_	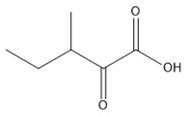	2.25	129.0542 (3.17)	68.9959
α-ketoisovalerate	C_5_H_8_O_3_	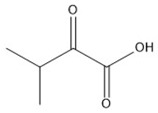	1.41	115.0391 (2.34)	59.0126

^a^ Figures in brackets represent the error of the precursor ion theoretical *m*/*z* and measured value (ppm).

**Table 2:** Recoveries (%) and limit of quantification (LOQ) for detection of the branched-chain keto acids in spiked samples using UPLC-Q-TOF/MS (*n* = 6).

Compounds	Recoveries (%)						LOQ	
	Serum (μmol/L)			Muscle (nmol/g)			Serum (μmol/L)	Muscle (nmol/g)
	10	20	50	1	2	5		
α-ketoisocaproate	114.3 (2.0) ^a^	84.3 (4.0)	86.8 (1.9)	85.9 (6.9)	93.6 (7.9)	94.7 (1.6)	0.06	0.09
α-keto-β-methylvalerate	98.7 (7.6)	100.2 (4.8)	99.0 (2.9)	95.3 (5.4)	103.2 (9.7)	100.8 (0.6)	0.09	0.12
α-ketoisovalerate	89.2 (3.2)	85.1 (3.8)	83.1 (1.2)	82.7 (1.8)	78.4 (1.0)	80.0 (7.1)	0.23	0.27

^a^ Figures in bracket represented relative standard deviation (%).

**Table 3:** Concentration of three BCKAs in animal samples (*n* = 6).

Compounds	Serum (μmol/L)	Muscle (nmol/g)
α-ketoisocaproate	27.63 ± 5.39	2.61 ± 0.89
α-keto-β-methylvalerate	18.80 ± 4.92	1.45 ± 0.48
α-ketoisovalerate	10.20 ± 3.94	1.24 ± 0.37

## References

[1] Tom A., Nair K.S. (2006). Assessment of branched-chain amino acid status and potential for biomarkers. J. Nutr..

[2] Lynch C.J., Adams S.H. (2014). Branched-chain amino acids in metabolic signalling and insulin resistance. Nat. Rev. Endocrinol..

[3] Lu J., Xie G., Jia W., Jia W. (2013). Insulin resistance and the metabolism of branched-chain amino acids. Front. Med..

[4] Holecek M., Siman P., Vodenicarovova M., Kandar R. (2016). Alterations in protein and amino acid metabolism in rats fed a branched-chain amino acid- or leucine-enriched diet during postprandial and postabsorptive states. Nutr. Metab..

[5] Hattori A., Tsunoda M., Konuma T., Kobayashi M., Nagy T., Glushka J., Tayyari F., Cskimming D.M., Kannan N., Tojo A. (2017). Cancer progression by reprogrammed BCAA metabolism in myeloid leukaemia. Nature.

[6] Wilkinson D.J., Hossain T., Hill D.S., Phillips B.E., Crossland H., Williams J., Loughna P., Churchward-Venne T.A., Breen L., Phillips S.M. (2013). Effects of leucine and its metabolite β-hydroxy-β-methylbutyrate on human skeletal muscle protein metabolism. J. Physiol..

[7] Wisniewski M.S.W., Carvalho-Silva M., Gomes L.M., Zapelini H.G., Schuck P.F., Ferreira G.C., Scaini G., Streck E.L. (2016). Intracerebroventricular administration of alpha-ketoisocaproic acid decreases brain-derived neurotrophic factor and nerve growth factor levels in brain of young rats. Metab. Brain Dis..

[8] Ananieva E.A., Van Horn C.G., Jones M.R., Hutson S.M. (2017). Liver BCATm transgenic mouse model reveals the important role of the liver in maintaining BCAA homeostasis. J. Nutr. Biochem..

[9] Wang X., Wei H., Cao J., Li Z., He P. (2015). Metabolomics analysis of muscle from piglets fed low protein diets supplemented with branched chain amino acids using HPLC-high-resolution MS. Electrophoresis.

[10] Zheng L., Wei H., Cheng C., Xiang Q., Pang J., Peng J. (2016). Supplementation of branched-chain amino acids to a reduced-protein diet improves growth performance in piglets: Involvement of increased feed intake and direct muscle growth-promoting effect. Br. J. Nutr..

[11] Rietman A., Stanley T.L., Clish C., Mootha V., Mensink M., Grinspoon S.K., Makimura H. (2016). Associations between plasma branched-chain amino acids, beta-aminoisobutyric acid and body composition. J. Nutr. Sci..

[12] Islam M.M., Nautiyal M., Wynn R.M., Mobley J.A., Chuang D.T., Hutson S.M. (2010). Branched-chain amino acid metabolon: Interaction of glutamate dehydrogenase with the mitochondrial branched-chain aminotransferase (BCATm). J. Biol. Chem..

[13] Cole J.T., Sweatt A.J., Hutson S.M. (2012). Expression of mitochondrial branched-chain aminotransferase and alpha-keto-acid dehydrogenase in rat brain: Implications for neurotransmitter metabolism. Front. Neuroanat..

[14] Lee A.J., Beno D.W.A., Zhang X., Shapiro R., Mason M., Mason-Bright T., Surber B., Edens N.K. (2015). A ^14^C-leucine absorption, distribution, metabolism and excretion (ADME) study in adult Sprague-Dawley rat reveals β-hydroxy-β-methylbutyrate as a metabolite. Amino Acids.

[15] Pimentel G.D., Rosa J.C., Lira F.S., Zanchi N.E., Ropelle E.R., Oyama L.M., Oller Do Nascimento C.M., de Mello M.T., Tufik S., Santos R.V.T. (2011). β-Hydroxy-β-methylbutyrate (HMβ) supplementation stimulates skeletal muscle hypertrophy in rats via the mTOR pathway. Nutr. Metab..

[16] Green C.R., Wallace M., Divakaruni A.S., Phillips S.A., Murphy A.N., Ciaraldi T.P., Metallo C.M. (2016). Branched-chain amino acid catabolism fuels adipocyte differentiation and lipogenesis. Nat. Chem. Biol..

[17] Escobar J., Frank J.W., Suryawan A., Nguyen H.V., Van Horn C.G., Hutson S.M., Davis T.A. (2010). Leucine and alpha-Ketoisocaproic acid, but not norleucine, stimulate skeletal muscle protein synthesis in neonatal pigs. J. Nutr..

[18] Columbus D.A., Fiorotto M.L., Davis T.A. (2015). Leucine is a major regulator of muscle protein synthesis in neonates. Amino Acids.

[19] Knerr I., Weinhold N., Vockley J., Gibson K.M. (2012). Advances and challenges in the treatment of branched-chain amino/keto acid metabolic defects. J. Inherit. Metab. Dis..

[20] Crowell P.L., Miller R.H., Harper A.E. (1988). Measurement of plasma and tissue-levels of branched-chain α-keto acids by gas-liquid chromatography. Method Enzymol..

[21] Fernandes A.A., Kalhan S.C., Njoroge F.G., Matousek G.S. (1986). Quantitation of branched-chain α-keto acids as their *N*-methylquinoxalone derivatives: Comparison of *O*- and *N*-alkylation versus-silylation. Biomed. Environ. Mass Spectrom..

[22] Pailla K., Blonde-Cynober F., Aussel C., De Bandt J.P., Cynober L. (2000). Branched-chain keto-acids and pyruvate in blood: Measurement by HPLC with fluorimetric detection and changes in older subjects. Clin. Chem..

[23] Hara S., Takemori Y., Yamaguchi M., Nakamura M., Ohkura Y. (1985). Determination of alpha-keto acids in serum and urine by high-performance liquid-chromatography with fluorescence detection. J. Chromatogr. B.

[24] Anumula K.R. (1995). Rapid quantitative-determination of sialic acids in glycoproteins by high-performance liquid-chromatography with a sensitive fluorescence detection. Anal. Biochem..

[25] Olson K.C., Chen G., Lynch C.J. (2013). Quantification of branched-chain keto acids in tissue by ultra fast liquid chromatography-mass spectrometry. Anal. Biochem..

[26] Yin B., Li T., Li Z., Dang T., He P. (2016). Sensitive analysis of 33 free amino acids in serum, milk and muscle by ultra-high Performance liquid chromatography-quadrupole-orbitrap high resolution mass spectrometry. Food Anal. Method.

[27] Thakare R., Chhonker Y.S., Gautam N., Alamoudi J.A., Alnouti Y. (2016). Quantitative analysis of endogenous compounds. J Pharm. Biomed. Anal..

[28] Kand’Ar R., Zakova P., Jirosova J., Sladka M. (2009). Determination of branched chain amino acids, methionine, phenylalanine, tyrosine and alpha-keto acids in plasma and dried blood samples using HPLC with fluorescence detection. Clin. Chem. Lab. Med..

[29] Cree T.C., Hutson S.M., Harper A.E. (1979). Gas-liquid chromatography of alpha-keto acids: Quantification of the branched-chain-alpha-keto acids from physiological sources. Anal. Biochem..

[30] Hutson S.M., Harper A.E. (1981). Blood and tissue branched-chain amino and alpha-keto acid concentrations-effect of diet, starvation, and disease. Am. J. Clin. Nutr..

